# The Critical Role of Lipid Metabolism in Health and Diseases

**DOI:** 10.3390/nu16244414

**Published:** 2024-12-23

**Authors:** Yucheng Zhu, Fangyan Wan, Jie Liu, Zhihao Jia, Tongxing Song

**Affiliations:** 1 College of Animal Science and Technology, Huazhong Agricultural University, Wuhan 430070, China; 2 Cambridge-Suda Genomic Resource Center, Suzhou Medical College, Soochow University, Suzhou 215000, China

Lipids make up 10–20% of the human body and serve as crucial energy storage substances as well as essential components of cell membranes. Physiologically, lipids are involved in regulating cellular structure and function, signaling pathways, and gene expression. Excess systemic free fatty acids (FFAs) and dietary lipids enter cells within non-adipose organs such as the liver, muscle, pancreas, and placenta, where they are deposited as ectopic fat, generating lipotoxicity. Chronic nutritional excess can lead to the accumulation of lipid metabolites, such as diacylglycerols, ceramides, and free fatty acids, which exacerbate lipid deposition and result in the onset of type 2 diabetes mellitus. Therefore, abnormal levels or the dysregulated accumulation of lipids within tissues are often associated with various diseases, including obesity, type 2 diabetes, non-alcoholic fatty liver disease, neurodegenerative diseases, infections, and cancer. Maintaining normal levels of lipid metabolism is crucial for overall health [1,2,3].

This Special Issue of Nutrients, entitled ‘Dietary Lipid, Adipose Tissue and Lipid Metabolism in Health and Diseases’, aims to highlight the significance of lipid metabolism in human health and disease. It seeks to elucidate the regulatory roles of key tissues and organs, including the intestine, liver, adipose tissue, muscle, pancreas, and placenta, in maintaining whole-body homeostasis.

Lipids are typically closely associated with clinical manifestations, and some can even directly reflect the clinical conditions [4,5]. In this Special Issue, we present several studies focusing on dysregulated lipid levels that contribute to the exacerbation of pathological conditions. Zhang et al. analyzed serum lipid levels from a dataset of 188,577 individuals of European descent to investigate the association with female infertility. The MR analysis revealed that low-density lipoprotein (LDL) cholesterol was significantly associated with the risk of infertility in women [6]. Concurrently, another study examined the alterations in serum lipid profiles, particularly the levels of polyunsaturated fatty acids, in 58 adult patients with psoriasis, increased the level of SFA and reduced oleic acid levels (*n*-9) that may contribute to the exacerbation of psoriasis [7]. Therefore, clarifying the underlying mechanisms of how lipids function in health and disease is essential for deepening our understanding of their roles.

Disruptions in lipid metabolism can significantly increase the risk of developing various diseases, including alcoholic liver disease and non-alcoholic fatty liver disease (NAFLD) [8]. Additionally, this Special Issue features research on how alcohol consumption interferes with the normal function of adipocytes. The study emphasized that under conditions of long-term alcohol consumption, the metabolic status of adipose tissue is significantly affected, facilitating lipolysis and leading to an excessive release of fatty acids, which in turn promotes the progression of alcoholic liver disease. This underscores the necessity for further research to elucidate the impediments to lipid deposition and transport caused by metabolic alterations in adipose tissue during the pathogenesis of fatty liver [9]. Interorgan crosstalk between major metabolic organs—adipose tissue, liver, and muscle—enhances lipid combustion, reduces lipid storage, and decreases free fatty acids in the vasculature, effectively ameliorating metabolic disorders [10].

Identifying key genes that regulate lipid metabolism is crucial for gaining a deeper understanding of how lipid metabolism contributes to disease pathogenesis. This insight, in turn, facilitates the establishment of therapeutic targets [11]. In addition, this Special Issue features research on alleviating metabolic disorders, highlighting key targets for the regulation of lipid metabolism. The study concludes that the role of PPARα as a nuclear transcription factor exerts a significant influence on diverse disease processes across multiple organs, including the liver and intestines [12]. This study reveals that liver-specific knockout of PPARα leads to elevated systemic levels of total cholesterol and low-density lipoprotein (LDL) cholesterol, resulting in liver damage and increased levels of inflammatory factors, leading to non-alcoholic steatohepatitis. In contrast, the intestine-specific knockout of PPARα significantly alleviates hepatic steatosis. Targeting PPARα may serve as a potential strategy for regulating metabolic disorders as a therapeutic agent. Proton-coupled amino acid transporters (Slc36As) are indispensable for brown adipose tissue and the systemic energy metabolism. Another study indicates that SLC36A2 is highly expressed in adipose tissue; the knockout of Slc36a2 results in increased energy expenditure and decreased lipid levels in both the liver and circulating blood [13]. Understanding the functions of key genes in the lipid metabolism is of significant importance for developing strategies to modulate these genes in alleviating metabolic disorders and obesity. 

In the Special Issue ‘Edition-Dietary Lipid, Adipose Tissue, and Lipid Metabolism in Health and Diseases’ we aimed to address the regulatory function of key tissues and organs, including the intestine, liver, adipose tissue, muscle, pancreas, and placenta, in whole-body homeostasis. These functions are mediated either directly through metabolic control or indirectly via multiple secreted factors. Further clarification that the onset of many critical diseases is directly associated with abnormal lipid metabolism underscores the significance of adipose metabolism. Revealing the mechanisms behind important fat metabolism disorders provides vital insights for improving metabolic health. Further exploration into the key genes that regulate lipid metabolism, particularly their roles in adipogenesis, thermogenesis, and other lipid metabolic processes, will pave the way for the identification of novel therapeutic targets. In summary, elucidating the associated relationships and uncovering causal links in lipid metabolism are crucial for the effective targeting of adipose metabolism to improve human health. 
